# Toward an intensive understanding of sewer sediment prokaryotic community assembly and function

**DOI:** 10.3389/fmicb.2023.1327523

**Published:** 2023-12-20

**Authors:** Jingjing Xia, Kai Yu, Zhiyuan Yao, Huafeng Sheng, Lijuan Mao, Dingnan Lu, HuiHui Gan, Shulin Zhang, David Z. Zhu

**Affiliations:** ^1^School of Civil & Environmental Engineering and Geography Science, Ningbo University, Ningbo, China; ^2^Institute of Ocean Engineering, Ningbo University, Ningbo, China; ^3^School of Marine Sciences, Ningbo University, Ningbo, China; ^4^Zhenhai Urban Planning and Survey Research Institute of Ningbo, Ningbo, China; ^5^Department of Civil and Environmental Engineering, University of Alberta, Edmonton, AB, Canada

**Keywords:** sewer sediment, prokaryotic community function, sulfur cycle, co-occurrence pattern, assembly mechanism

## Abstract

Prokaryotic communities play important roles in sewer sediment ecosystems, but the community composition, functional potential, and assembly mechanisms of sewer sediment prokaryotic communities are still poorly understood. Here, we studied the sediment prokaryotic communities in different urban functional areas (multifunctional, commercial, and residential areas) through 16S rRNA gene amplicon sequencing. Our results suggested that the compositions of prokaryotic communities varied significantly among functional areas. *Desulfomicrobium*, *Desulfovibrio*, and *Desulfobacter* involved in the sulfur cycle and some hydrolytic fermentation bacteria were enriched in multifunctional area, while *Methanospirillum* and *Methanoregulaceae*, which were related to methane metabolism were significantly discriminant taxa in the commercial area. Physicochemical properties were closely related to overall community changes (*p* < 0.001), especially the nutrient levels of sediments (i.e., total nitrogen and total phosphorus) and sediment pH. Network analysis revealed that the prokaryotic community network of the residential area sediment was more complex than the other functional areas, suggesting higher stability of the prokaryotic community in the residential area. Stochastic processes dominated the construction of the prokaryotic community. These results expand our understanding of the characteristics of prokaryotic communities in sewer sediment, providing a new perspective for studying sewer sediment prokaryotic community structure.

## 1 Introduction

Urban sewer system, commonly known as “the blood vessel of a city”, serves sewage collection and transportation functions, but also is a “biochemical reactor” that can change the form of pollutants, responsible for many important ecological functions (Mathioudakis and Aivasidis, 2009), serving as a reflection of urban ecology. Different from natural environments such as soil/water which have been around for millions of years, the sewer system is an artificial environment and is a comparatively new habitat for microorganisms (McLellan and Roguet, 2019). The feature of the prokaryotic community in sediment has caught the attention of environmental microbiologists.

Most previous studies have used different types of molecular tools to study specific sediment microorganisms (e.g., sulfur-oxidizing, sulfate-reducing, and methanogenic bacteria) in sewer systems (Dong et al., 2017; Shi X. et al., 2020). There are fewer comprehensive studies on the composition and functional characteristics of prokaryotic communities in sewer sediments. In recent years, high-throughput sequencing technologies have dramatically deepened our insights into prokaryotic communities in diverse ecosystems (Song et al., 2023; Yang et al., 2023). Jin et al. (2018) preliminarily explored the prokaryotic succession patterns in biofilms along urban sewage networks and explored the relationship between sewage quality and prokaryotic communities. However, most sewer sediment studies were conducted using lab-scale reactors (Ren et al., 2022; Sharma et al., 2023), which failed to accurately replicate the intricate environmental conditions in actual sewer systems. In different urban functional areas, the sediment physicochemical properties showed regular change due to the various sources of sewage which likely led to prokaryotic community change. The sewage and sewer sediment properties are highly related to land use patterns (Ekklesia et al., 2015; Maritz et al., 2019). As a result, we assume that functional area could be an important factor that shapes the prokaryotic community distribution and functions in sewer sediment. Since various sewer systems demand different management strategies, understanding the prokaryotic variance across different functional areas could contribute to precision management, improving sewer quality and health.

Microbes do not exist in isolation and often establish an intricate inter-species web that regulates the function and structure of ecosystems (Freilich et al., 2010). Analyzing the co-occurrence patterns could offer fresh perspectives on the sediment habitat and enhance our comprehension of the sediment prokaryotic community, going beyond considerations of diversity and composition (Barberán et al., 2012; Faust and Raes, 2012). Although a wide array of studies revealed the prokaryotic community co-occurrence pattern in complex sediment environments ranging from river to ocean (Djurhuus et al., 2020; Zhang et al., 2021), there is still a knowledge gap remains regarding the sewer sediment, a special artificial habitat for prokaryotic co-occurrence. Intense debates and discussions have surrounded this enduring issue concerning community assembly mechanisms. Both stochastic processes (including homogenizing dispersal, dispersal limitation, and drift) and deterministic processes (including homogeneous selection and heterogeneous selection) facilitate the assembly of prokaryotic communities in diverse habitats. However, it is not yet clear how the sediment prokaryotic community assemblies vary with the functional areas.

Here we hypothesized that functional area could be a significant factor that shapes the prokaryotic community distribution and functions in sewer sediment. Physicochemical properties may impose different stresses that drive community assembly processes, thereby shifting the prokaryotic community structure in the sediment. To address the research needs discussed above and to provide an improved basis for future sewer sediment monitoring efforts, we profiled prokaryotic communities in sewer sediments from typical urban functional areas, including multifunctional, commercial, and residential areas. This study aims to (1) characterize the prokaryotic diversity and composition of sewer sediment; (2) elucidate the relationship between prokaryotic community structure and physicochemical properties; (3) uncover the prokaryotic co-occurrence and assembly patterns in the sewer sediment in different functional areas. This study aims to comprehensively provide insights into prokaryotic communities in different functional areas, and provide new insights for the analysis of urban sewer sediment prokaryotic communities.

## 2 Materials and methods

### 2.1 Sample collection

Typical multifunctional, commercial, and residential areas were selected in Ningbo, China. In each functional area, two separate main sewer systems were randomly selected. Under the expert guidance of the Zhenhai Urban Planning and Survey Research Institute of Ningbo, we selected an area in the city where residential buildings were relatively concentrated as a residential area, a district where retail businesses were clustered and transactions were frequent as a commercial area, and a district with multiple functions, such as residential, commercial, service, and administrative areas, as a multifunctional area. A total of 15 sampling sites were strategically designated, spanning three distinct functional areas. The specific information on sewer is detailed in Supplementary Table 1. The in-field criteria are as described by Crabtree (1989). There was a two-week drying period before sampling. Determination of temperature, dissolved oxygen, and pH of sewage at different sampling sites using the Multiparameter Instrument (Pro-Plus, Xylem, America). Additionally, the hydrogen sulfide concentrations in the manhole were measured using the Hydrogen sulfide gas recorder (H_2_S Gas Monitor (PPM) gas monitor, Acrulog™, Australia). Sediments were collected in quadruplicate using a shovel. The 60 collected samples (fifteen sites by four replicates) were carefully placed into sterile plastic bags with airtight seals. These bags were stored in ice packs at controlled low temperatures to maintain sample integrity. After transportation, sediments were preserved at 4°C for sedimentary physicochemical property analysis and at −80°C for DNA isolation, respectively.

### 2.2 Physicochemical analysis

The sediment metrics including sediment pH, total organic carbon (TOC), total phosphorus (TP), total nitrogen (TN), nitrate-nitrogen (NO_3_^–^-N), ammonium-nitrogen (NH_4_^+^-N), and available sulfur (AS) contents, total solid (TS), volatile solid (VS), and physical texture (sand, silt, and clay contents) were measured. Determination of pH in sediments using a pH meter: water (1:5) extract. Sediment TOC and TN were assessed with TOC analyzer (multi-N/C3100, analytic jena, Germany). Sediment TP was measured using a digestion method. Sediment NH_4_^+^-N and NO_3_^–^-N were determined by the method described in the Chinese Committee of Agricultural Chemistry (1983). Sediment AS was determined by the BaSO_4_ turbidimetric method. Sediment TS was measured by high-temperature burning weighing method and sediment VS was measured by the Gravimetric method. Sediment physical texture was measured by a laser diffraction particle size analyzer (2000LD, Bettersize, China). The physicochemical properties of each sediment sample were obtained from four independent replicate samples. The temperature, pH, and dissolved oxygen of sewage are detailed in Supplementary Table 2.

### 2.3 DNA extraction, PCR amplification and sequencing

The PowerSoil^®^ DNA kit (MoBio Laboratories, Carlsbad, CA, USA) was used to isolate prokaryotic DNA from each sample. For the prokaryotic community, we used the universal primers 515F (5′-GTGCCAGCMGCCGCGGTAA-3′) and 806R (5′-GGACTACNVGGGTWTCTAAT-3′) to amplify the V4 region of the 16S rRNA gene. Next, the amplified and cleaned DNA samples were sequenced using the pairwise end-to-end (2 × 300 bp) method by an Illumina MiSeq platform (Illumina, Inc., San Diego, CA, USA). The raw sequences were submitted to the NCBI BioProject database. The accession number was PRJNA1015661.

### 2.4 Illumina data processing

Use Usearch fastq_mergepairs command with default parameters in USEARCH v.11 software to combine paired-end reads (Edgar, 2010). In brief, redundant sequences and chimeras are filtered using the USEARCH unoise3 algorithm. USEARCH fastx_uniques was used to sort the retained clean markers in non-redundant abundance order. We classified sequences with ≥97% similarity as zero-radius operational taxonomic units (ZOTUs) (Edgar, 2010). The sequences with the highest abundance and coverage in each ZOTU were selected as representative sequences and compared with the Greengenes Database (release 13.8) using PyNAST to get taxonomic information of each ZOTU (DeSantis et al., 2006; Caporaso et al., 2010).

### 2.5 Statistical analysis

Calculation of alpha diversity indices of the sediment prokaryotic communities used the “vegan” package (Dixon, 2003). Differences in prokaryotic community samples between functional areas were analyzed by principal coordinate analysis (PCoA). The significance of differences was tested by Permutational multivariate analysis of variance (PERMANOVA). Linear discriminant analysis effect size (LEfSE) was used to identify the taxa with significant differences across functional areas (Chen H. et al., 2021). The program “functional annotation of prokaryotic taxa” (FAPROTAX) was used to predict the environmental biochemical functions of the prokaryotic communities in sediment. The effect of physicochemical properties on prokaryotic community composition was investigated using redundancy analysis (RDA). The two-way correlation network analysis was conducted using the 200 most abundant genera in terms of taxonomic abundance and physicochemical properties (performed using Network and Gephi). The correlation matrix was constructed by performing the Spearman’s rank correlation coefficient for each pairwise comparison (with a threshold of Spearman coefficient ≥0.6 and *p* < 0.05).

### 2.6 Co-occurrence network and community assembly analysis

Co-occurrence network analysis was based on Spear correlation (Spearman | R| > 0.9, *p* < 0.01), occurring in more than 80% of the samples. Subsequently, the prokaryotic co-occurrence network was established in Gephi (0.9.7). The role of each node in the network is identified by the inter-module connectivity (Pi) and intra-module connectivity (Zi) of the module attributes (Guimerà and Nunes Amaral, 2005). The “keystone species” refers to nodes determined to be network hub and module hub based on intra-module connectivity (Zi) and inter-module connectivity (Pi) values. The neutral community model was predicted by the “Hmisc” package. The iCAMP method was used to determine the assembly process (Ning et al., 2020).

## 3 Results

### 3.1 Sediment physicochemical properties and prokaryotic community diversity

**FIGURE 1:**
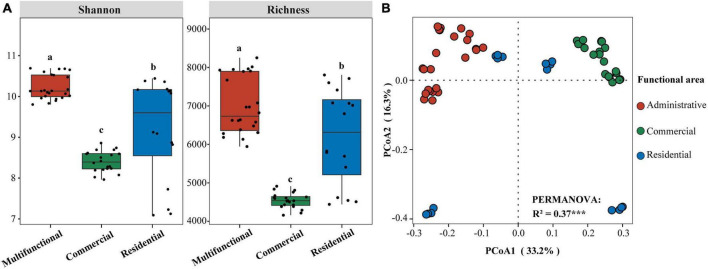
**(A)** Differences of Shannon and Richness indices in sediment from different functional areas. Significance of differences is indicated by “a, b, c” (*p* < 0.05; multiple comparison with ANOVA tests); **(B)** Principal coordinate analysis (PCoA) based on Bray-Curtis dissimilarity and PERMANOVA tests showing the variation of sediment prokaryotic communities from different functional areas. ***p < 0.001.

Sewer sediment physicochemical properties fluctuated dramatically across functional areas (Supplementary Figure 1). The hydrogen sulfide (H_2_S) concentration in the multifunctional area (7.69 ppm) was found to be significantly higher compared to the commercial area (3.06 ppm) and the residential area (3.2 ppm) (*p* < 0.05). Moreover, the multifunctional area exhibited the highest content of available sulfur (AS), total nitrogen (TN), total phosphorus (TP), and total organic carbon (TOC) while the residential area had the highest level of nitrate-nitrogen (NO_3_^–^-N) (Supplementary Figure 1). The pH was higher in the commercial area compared to the multifunctional area and residential area (Supplementary Figure 1). At the ZOTU level, the functional area significantly affected the diversity of sediment prokaryotic communities (Figure 1A). The Shannon and Richness indices were significantly different among functional areas (*p* < 0.05). Shannon index was the highest in multifunctional area, followed by residential area and commercial area. Similarly, the Richness index showed the same phenomena. Additionally, the random forest (RF) analysis identified TS (total solid), TOC (total organic carbon), and TP (total phosphorus) as relatively important variables in regulating the diversity of sediment prokaryotic communities (Supplementary Figure 2). Differences in prokaryotic communities in sewer sediments from three functional areas were analyzed using the principal coordinate analysis (PCoA) (Figure 1B). Prokaryotic community composition differed significantly among functional areas (*p* < 0.001) (Figure 1B).

### 3.2 Variation of sediment prokaryotic community composition and function

**FIGURE 2:**
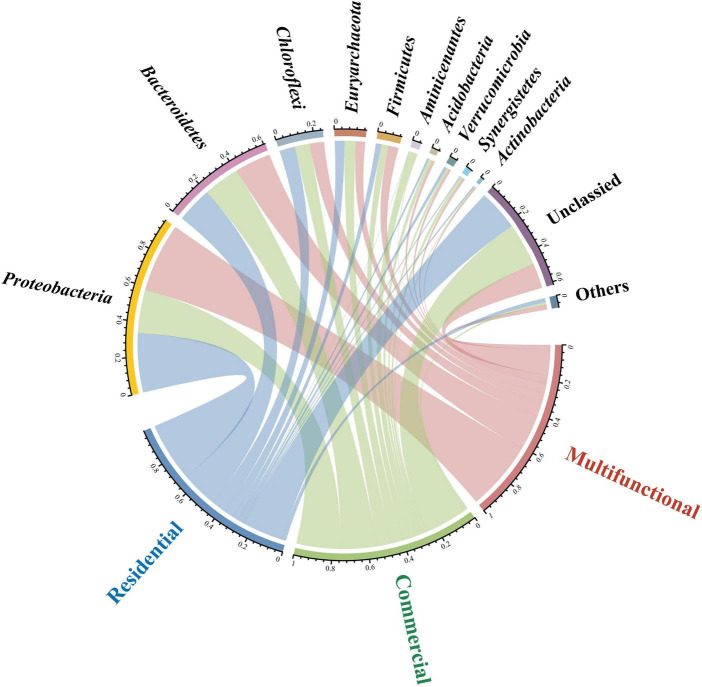
Circos plot showing the distribution of sewer sediment prokaryotic community composition at phylum level in different functional areas.

Circos plot uncovered the distribution of sewer sediment prokaryotic community composition in different functional areas at the phylum level (Figure 2). *Proteobacteria* and *Bacteroidetes* were the most abundant phyla in the sediment. *Proteobacteria* exhibited significantly higher abundance in the multifunctional sediment (*p* < 0.05), whereas the relative abundance of *Aminicenantes* was found to be the highest in the commercial area. Linear discriminant analysis (LDA) was used to identify the differentially abundant taxa among different functional areas (Supplementary Figure 3). Interestingly, various functional taxa were identified as significantly discriminant taxa in different functional areas. *Desulfomicrobium*, *Desulfovibrio*, and *Desulfobacter* (Wang et al., 2023) are involved in the sulfur cycle, and some hydrolytic fermentation bacteria, such as *Trichococcus*, *Ornatilinea*, and *Anaerolinea* (Jin et al., 2018), were enriched within the prokaryotic communities in the multifunctional area. In addition, *Methanospirillum* and *Methanoregulaceae* (Qian et al., 2023), which were related to methane metabolism were significantly discriminant in the commercial area.

We conducted further analysis to investigate the distribution of methanogenic archaea (MA) and sulfate-reducing bacteria (SRB) in sediments across various functional areas. The relative abundances of SRB in the multifunctional, commercial, and residential areas were quantified as 1.96, 1.05, and 0.91%, respectively. Remarkably, the multifunctional area exhibited a comparatively higher relative abundance of SRB. Furthermore, we identified four primary genera, namely *Methanothrix*, *Methanobacterium*, *Methanospirillum*, and *Methanomassiliicoccus*, as the predominant members of the accumulated MA community. The relative abundances of MA were determined to be 3.58% in the multifunctional area, 4.73% in the commercial area, and 3.44% in the residential area. These findings indicated that the commercial area has the highest relative abundance of MA.

**FIGURE 3:**
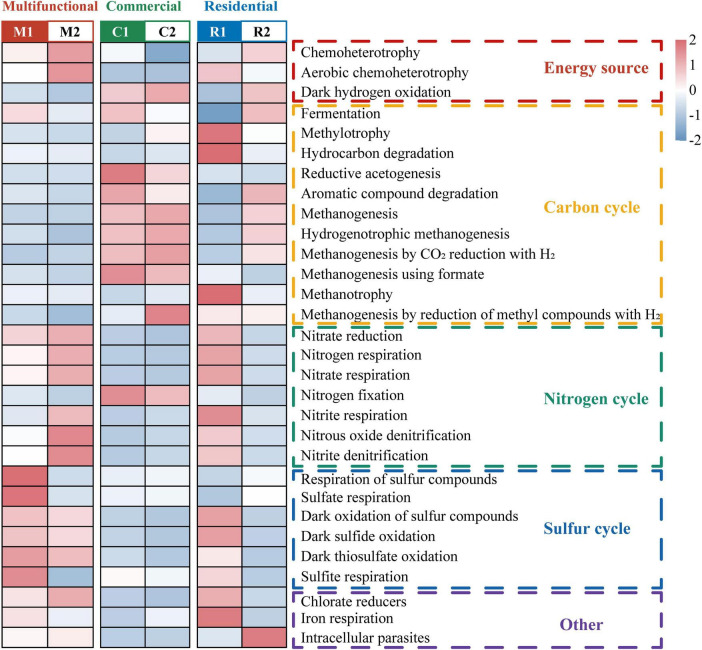
Heatmap representing major differences in predicted functions among sewer sediments from different functional areas based on Functional annotation of prokaryotic taxa (FAPROTAX).

The ecological functions of prokaryotic communities present in the sewer sediment were characterized by annotating them into 76 functional groups and categorized into 5 major groups including carbon cycle, nitrogen cycle, sulfur cycle, energy source, and other predicted functions using “functional annotation of prokaryotic taxa” (FAPROTAX) (Figure 3). Based on principal coordinate analysis (PCoA), there was an obvious separation among functional areas (Supplementary Figure 4). Permutational multivariate analysis of variance (PERMANOVA) further revealed that significant differences were observed in the prokaryotic community functions among functional areas (*p* < 0.05). The top 30 most abundant functions were identified and presented as a heatmap (Figure 3). According to the results, chemoheterotrophy, methanogenesis, and fermentation were the most common functions, and multiple sulfur metabolism-related function genes, including dark oxidation of sulfur compounds, respiration of sulfur compounds, and sulfate respiration were also observed. Interestingly, we observed distinct patterns in the functional group relative abundance associated with different ecological functions (Figure 3). Specifically, the multifunctional area exhibited the highest relative abundance of functions related to the sulfur cycle. On the other hand, functional groups associated with methanogenesis, the process involved in methane production, were concentrated in the commercial area.

### 3.3 Physicochemical properties affecting the prokaryotic community

**FIGURE 4:**
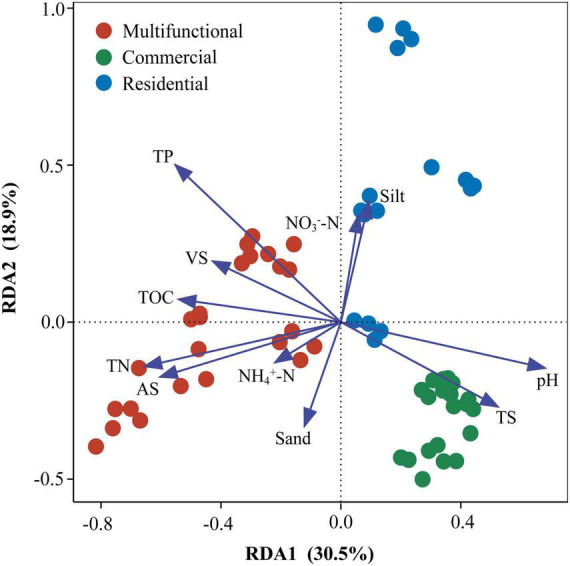
Redundancy analysis (RDA) of sewer sediment prokaryotic communities with physicochemical properties. TP, total phosphorus; TN, total nitrogen; AS, available sulfur; TS, total solid; TOC, total organic carbon; VS, volatile solid; NO_3_^–^-N, nitrate- nitrogen; NH_4_^+^-N, ammonium-nitrogen; pH, sediment pH; physical texture (sand, and silt contents).

**FIGURE 5:**
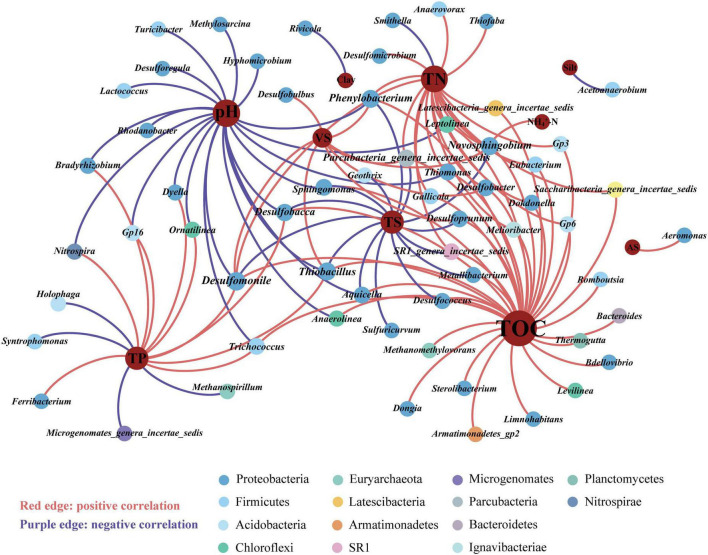
Two-way correlation network analysis between prokaryotic genera and sediment physicochemical properties. Different colors of nodes were classified into various phyla. TOC, total organic carbon; pH, sediment pH; TP, total phosphorus; TN, total nitrogen; TS, total solid; VS, volatile solid; AS, available sulfur; NH_4_^+^-N, ammonium-nitrogen; physical texture (sand, and silt contents).

Redundancy analysis (RDA) revealed that the prokaryotic community structure was tightly related to physicochemical properties (Figure 4). The first axis (RDA1) and second axis (RDA2) explained 49.4% of differences in the community structure. TP (*R*^2^ = 0.51, *p* < 0.01), TN (*R*^2^ = 0.36, *p* < 0.01), and pH (*R*^2^ = 0.41, *p* < 0.01), were significantly related to the sediment prokaryotic community (Supplementary Table 3). Two-way correlation network analysis was conducted to further demonstrate the correlation between sediment physicochemical properties and genus-level prokaryotic communities (Figure 5). TOC, pH, and TN stood for the key hub nodes in the network, showing complex connections with prokaryotes. TOC and TN were positively correlated with most prokaryotes. On the contrary, pH and TS showed negative correlations with most prokaryotes.

Given the disparities in the distribution of methanogenic archaea (MA) and sulfate-reducing bacteria (SRB) in various functional areas, we further investigated their correlation with physicochemical properties. Spearman correlation analysis revealed that the response sensitivity of MA and SRB to physicochemical properties in sewer sediments was different (Supplementary Figure 5). Most SRB was positively correlated with total nitrogen (TN), total phosphorus (TP), total organic carbon (TOC), nitrate-nitrogen (NO_3_^–^-N), ammonium-nitrogen (NH_4_^+^-N), available sulfur (AS), and volatile solid (VS), while negatively correlated with pH and total solid (TS). *Methanothrix*, which was the most abundant genus of MA, was positively correlated with total organic carbon (TOC), total phosphorus (TP), and volatile solid (VS). In contrast, it was negatively correlated with nitrate-nitrogen (NO_3_^–^-N) and total solid (TS).

### 3.4 Co-occurrence pattern and keystone species in sediment prokaryotic communities

**FIGURE 6:**
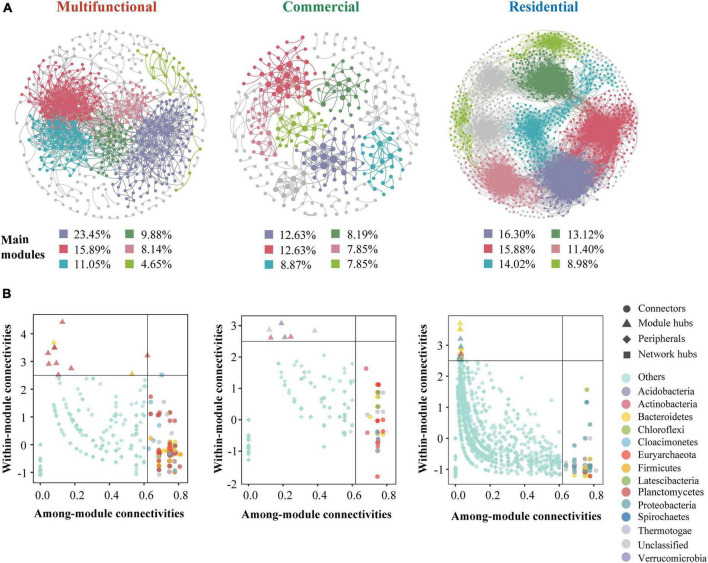
Co-occurrence network analysis of the sewer sediment prokaryotic communities from different functional areas. **(A)** Co-occurrence networks in different functional areas. **(B)** keystone species analysis.

The co-occurrence patterns of prokaryotic ZOTUs (at least 80%) were visualized by network analysis (Figure 6A). More positive edges were detected in all networks, implying that prokaryotic ZOTUs tended to cooperate and coexist rather than compete in the sewer sediment. The topological parameters were summarized as shown in Table 1. The node numbers, edge numbers, and average degree which can assess sediment prokaryotic network complexity were the highest in the residential sediment, whereas the lowest in the commercial area sediment. On the contrary, modularity and positive proportion were the highest in the commercial sediment. The potential topological roles of taxa in the networks were assessed according to within-module connectivity (Zi) and among-module connectivity (Pi) values:(1) network hub (Zi > 2.5 and Pi > 0.62); (2) module hub (Zi > 2.5 and Pi ≤ 0.62); (3) connector (Zi ≤ 2.5 and Pi > 0.62); and (4) peripheral (Zi ≤ 2.5 and Pi ≤ 0.62) (Zhang et al., 2023; Figure 6B). *Thiobacillus*, *Methanospirillum*, and *Geobacter* were keystone species with the highest frequency of occurrence in multifunctional, commercial, and residential areas, respectively. In the multifunctional area, 1 network hub (ZOTU275, belonging to *Anaerolineaceae*) was recognized. More specifically, 152 keystone species including 140 connectors, 11 module hubs, and 1 network hub were identified in the multifunctional sewer network, which mainly belongs to *Thiobacillus*, *Desulfovibrio*, and *Methanobacterium*. In the commercial area, we identified a total of 63 connectors and 6 module hubs, which mainly belonged to *Methanospirillum*, *Holophaga*, and *Methanothrix*. In the residential area, keystone species mainly belonged to *Geobacter*, *Syntrophorhabdus*, and *Candidatus_Cloacamonas*.

**TABLE 1:** Topological properties of the sewer sediment co-occurrence network from different functional areas.

Functional areas	Multifunctional	Commercial	Residential
Nodes	516	293	1448
Edge	1385	459	19530
Average degree	5.368	3.133	26.975
Modularity	0.6	0.817	0.72
Percentage of negative correlations	29.24	24.18	32.7
Percentage of positive correlations	70.76	75.82	67.3
Graph density	0.01	0.011	0.019
Clustering coefficient	0.342	0.376	0.511
Average path length	5.058	6.897	5.435

### 3.5 Assembly mechanisms across sewer sediment in different functional areas

**FIGURE 7:**
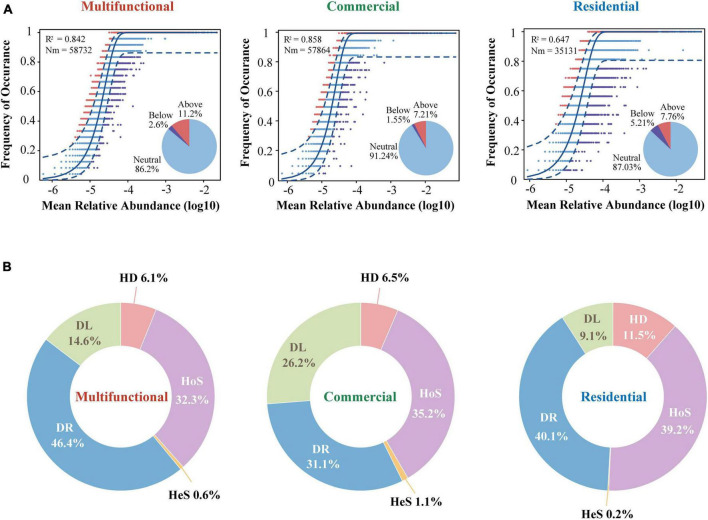
The assembly process of sewer sediment prokaryotic communities from different functional area. **(A)** The neutral community model. The Blue dashed line indicates 95% confidence intervals for the prediction of neutral model. *R*^2^ values indicate the goodness of fit. The Nm parameter reflects immigration times metacommunity size. **(B)** The contributions of different ecological processes in assembling the prokaryotic community. HeS, heterogeneous selection; HoS, homogeneous selection; HD, homogenizing dispersal; DL, dispersal limitation; DR, drift.

Considering that the composition and diversity of sewer sediment prokaryotes were spatially dynamic, we investigated the community assembly mechanisms driving forces that shaped the community structure, which could provide the theoretical basis for sediment prokaryotic community regulation. The neutral model fitted the data of all three functional areas, producing the lowest *R*^2^ (0.65) in residential area and the highest *R*^2^ (0.86) in commercial area (Figure 7A). The majority of dots were in the neutral range (86.2% ∼ 91.2%), suggesting that stochastic processes dominated the prokaryotic community assembly in sewer sediments. Mitigation rates were higher in the multifunctional area (*m* = 0.82) and the commercial area (*m* = 0.82) than in the residential (*m* = 0.49) area, suggesting that the multifunctional area and commercial area were less limited by dispersal (Figure 7A). Among the three functional areas, the neutral part was consistently dominant. In general, the result of the neutral model suggested that the processes of random dispersal and ecological drift were more significant than selection in assembling the prokaryotic community in different functional areas.

In addition, we quantified the relative contribution of major ecological processes that structure the prokaryotic community in the sediments. The ecological processes mainly include stochastic and deterministic processes. The stochastic processes included homogenizing dispersal, dispersal limitation, and drift, while the deterministic processes were divided into heterogeneous selection and homogeneous selection. Generally, our findings reveal that stochastic processes significantly dominate the assembly of the prokaryotic community, as shown in Figure 7B. Homogeneous selection was the highest in the residential area sediments (39.2%) (Figure 7B). Conversely, the importance of dispersal limitation was lowest in residential sediments (9.1 and 0.2%, respectively) (Figure 7B).

## 4 Discussion

### 4.1 Sediment prokaryotic diversity varies with functional area

Our research revealed that, compared to various natural sediment ecosystems, the Shannon index of the sewer sediment was notably higher (Zhang et al., 2021; Wang et al., 2022). This might be attributed to the varying environmental conditions between artificial pipes and natural ecosystems. The organic matter from sewage might be decomposed into numerous compounds (Shi et al., 2018; McLellan and Roguet, 2019), leading to the increased species richness and diversity of the sewer sediment prokaryotic community. In addition, the existence of foreign matter such as fibers and plastics can create extra microhabitats and ecological niches that afford diverse microorganisms the chance to establish and flourish (Wright et al., 2020), thereby contributing to overall diversity. Furthermore, the rapid flow velocity of sewage creates a distinct physical environment compared to natural ecosystems. The constant movement and turbulent flow of wastewater could influence the distribution of microorganisms and nutrient availability within the sediment (Gudjonsson et al., 2002), further contributing to the increased diversity observed in sewer sediment compared to natural ecosystems.

Moreover, the prokaryotic diversity values (Shannon and Richness indices) were significantly higher in the multifunctional area sediments (Figure 1A), while they were significantly correlated with total solid (TS), total organic carbon (TOC), total phosphorus (TP), and volatile solid (VS) (Figure 4). In the multifunctional area, higher levels of total phosphorus (TP) and total organic carbon (TOC) were observed compared to the commercial and residential areas (Supplementary Figure 1). These carbon and phosphorus components are essential nutrients for the growth and reproduction of prokaryotic communities (Wang et al., 2020; Yi et al., 2021), which leads to a higher α-diversity of prokaryotic communities in multifunctional areas. In addition, the β-diversity analysis yielded a distinct clustering of the samples (Figure 1B). This finding is consistent with a previous study on urban river sediments, which also showed similar clustering patterns based on functional area types (Wang et al., 2021). In urban sewer system, sewage is an important source for the sediment community, so the various sources of sewage might contribute to the different sediment prokaryotic structures of each functional area (Figure 1B). Indicating that the sewer sediment of each functional area owned its unique microbiome.

### 4.2 Dominant prokaryotic assemblages and functions are altered with distinct sewer habitats

Consistent with observations from previous studies, the prokaryotic communities in sewer sediment were dominated by *Proteobacteria* and *Bacteroidetes* (Figure 2). However, this is different from natural habitats, which were mainly dominated by *Actinobacteria* and *Proteobacteria* (Zhang et al., 2021). The sewerage contains a high concentration of pollutants, such as organic matter, nutrients, and heavy metals. The physicochemical properties of the sewerage, including low pH value and dissolved oxygen levels, were significantly different from natural environments (McLellan and Roguet, 2019). These differences may result in changes in sewer sediment prokaryotic composition. Additionally, distinct sewer sediment prokaryotic community compositions were observed among functional areas. *Desulfomicrobium*, *Desulfovibrio*, and *Desulfobacter*, typical sulfate-reducing bacteria (SRB), were more abundant in the multifunctional area sediment (Supplementary Figure 3). Some significantly discriminant taxa remain in the multifunctional area sediment, including fermentation bacteria (FB) and hydrogen-producing acetogen (HPA) (Supplementary Figure 3). The hydrolysis and fermentation processes produce volatile fatty acids and macromolecular acids, which provide appropriate conditions for the SRB (Jin et al., 2015), which may lead to a higher relative abundance of SRB, ultimately leading to relatively high hydrogen sulfide concentrations in multifunctional areas. Functional annotation of prokaryotic taxa (FAPROTAX) further demonstrated that functions associated with sulfur cycling were particularly notable in the multifunctional areas (Figure 3). Combined with in-field measurements of hydrogen sulfide concentrations, we should pay attention to the hydrogen sulfide risk in multifunctional area sewers. Recently, amounts of studies have reported methane production from freshwater sewerage systems, implying that sewer sediment is an important greenhouse gas contributor to the environment (Chen et al., 2020; Chen S. et al., 2023). Our work revealed that *Methanospirillum, and Methanoregulaceae*, associated with methane production, were more abundant in the commercial area sediments (Supplementary Figure 3). Moreover, *Syntrophomonas* and *Smithella* also had a higher relative abundance in commercial area (*p* < 0.05). It was reported that they can form powerful symbiotic interactions with methanogens (McInerney et al., 1981; Embree et al., 2013). Meanwhile, the functional capacity for methane metabolism was also predominantly concentrated in the commercial area (Figure 3). Thus, we speculated that sewer sediments in commercial areas may obtain higher potential in methanogenesis.

### 4.3 Local physicochemical properties shape the prokaryotic distribution

Environmental factors play a crucial role in the formation of microbial community composition and functional characteristics (Nelson et al., 2016; Gibbons, 2017; Louca et al., 2017). It has been demonstrated that the structure of natural sediment prokaryotic communities is correlated with TN, TOC pH, etc., (Bao et al., 2023; Feng et al., 2023). Our results revealed that sediment TN, TP, TOC, and pH were the major elements strongly related to the structure of the prokaryotic community (Figures 4, 5). Nutrient levels affect the growth of prokaryotic communities in sediments. In most sediment ecosystems, TN and TP serve as the main limiting nutrients and play an essential role in regulating prokaryotic community structure (Cai et al., 2022; Chen Y. et al., 2023). Similarly, TOC, as a source of energy and carbon for microbial metabolism, also influences the composition, diversity, and function of microbial communities (Hu et al., 2022). The significance of pH in forming microbial communities has been widely documented across a variety of ecosystems (Fierer and Jackson, 2006; Jones et al., 2019; Naz et al., 2022). It affects the various life processes of aquatic organisms by influencing cellular osmotic pressure, enzyme synthesis, and nutrient uptake (Santini et al., 2022). A large number of studies have revealed that pH could change nutrient effectiveness and organic carbon content, thus directly or indirectly affecting the diversity, composition, and abundance of microbial communities (Colman et al., 2016; Dong et al., 2022). In total, these physicochemical properties were demonstrated to a crucial role in forming prokaryotic communities. Nonetheless, this study may not provide a complete explanation for all the observed changes. Therefore, more comprehensive investigations are needed to explore the potential influence of other factors.

### 4.4 The co-occurrence networks revealed a more complex co-occurrence network in sewer sediment from residential area

Microbial networks have been proven to be an efficient method for revealing microbial co-occurrence patterns (Qiu et al., 2021). In terms of global network topology, the findings indicated that the residential area network was characterized by a higher level of complexity and a lower proportion of positive correlations (Figure 6A and Table 1). Conversely, the commercial area characterized by the simplest network exhibited the highest level of positive correlations (Figure 6A and Table 1). Previous studies indicated that an increase in complexity is generally accompanied by a decrease in positive correlation proportion (Li and Xiao, 2023), which is consistent with our study. Complexity leads to stability, suggesting a robust correlation between ecological stability and the complexity of microbial networks (Okuyama and Holland, 2008). Higher network complexity increases inter-specific interaction, providing microbial communities with favorable characteristics for effective resistance to environmental interference (Konopka et al., 2015; Jiao et al., 2020), which leads to a significant increase in the stability of microbial ecosystems. We speculated the possible reason was that the sources and characteristics of the sediment in residential area were relatively homogeneous. Generally, in more homogeneous environments, the composition of prokaryotic communities tends to be more stable, which favors the formation of a complex and stable network. In contrast, commercial area sewage was always characterized by a large sewage flow rate, complex water quality, and significant water quality fluctuations. These implied that the composition of prokaryotes in commercial areas sediment might be variable, leading to an unstable network. Previous studies indicated that positive correlations represent cooperative relationships and can enhance or maintain community stability and diversity through ecological niche complementation or synergism (Coyte et al., 2015). Therefore, prokaryotic communities in commercial area tend to cluster forming a more cooperative network to transfer information and share resources, to enhance the stability of the network.

The key module hubs or nodes may be important members of the prokaryotic community in a network, playing a vital role in sustaining community stability (Shi Y. et al., 2020). It has been demonstrated that keystone species have more significant roles in sustaining network structure than the other microbes, and the deprivation of these key taxa may contribute to the dissolution or even disintegration of the network (Shi Y. et al., 2020; Liu et al., 2022). *Anaerolineaceae*, which as the representative group of the *Chloroflexi*, could degrade carbohydrates (e.g., amino acids) (Zhang et al., 2017), was the network hub in the multifunctional area network. The highest occurrence of keystone species in multifunctional area network is *Thiobacillus* and *Desulfovibrio*, which are involved in nitrogen metabolism and sulfur biogeochemical cycling (Chen et al., 2018; Li et al., 2023). *Desulfovibrio*, a typical sulfate-reducing bacterium, was found to be a keystone species in the multifunctional area network, indicating that this network has specific ecological and geochemical functions related to sulfate cycling, such as sulfide generation. *Geobacter* was the keystone species of the residential area networks. *Geobacter* is an oligotrophic bacterium that can transfer electrons to facilitate the degradation of the chemical compound (Fan et al., 2019). In addition, the keystone species of the commercial area such as *Methanospirillum* were reported to be involved in methanogenesis, which could convert certain compounds from organic waste and wastewater into methane. This implied that the emission of greenhouse gases from commercial areas ought to be given greater consideration.

### 4.5 Stochastic processes dominate prokaryotic community assembly in sewer sediment

A key target in microbial ecology is quantifying the relative contribution of deterministic and stochastic processes in microbiome assembly (Weigel et al., 2023). In different ecosystems, the relative importance of deterministic and stochastic processes seems to be different (Matar et al., 2017; Huo et al., 2023; Lin et al., 2023). Considering that microbial diversity and community composition vary across functional areas, we further investigated the microbial assembly mechanisms. In this study, the stochasticity process dominated the prokaryotic community assembly across the three functional areas. Previous studies showed that stochastic processes largely dominate the assembly of microbial communities in some urban wastewater infrastructure (Wu et al., 2019), while deterministic processes were relatively more important in some natural ecosystems (Chen Q. et al., 2021; Lu et al., 2023). Natural ecosystems are typically relatively stable and undisturbed systems, while engineered ecosystems are typically dynamic and constantly changing systems that require adaptation to changing environmental conditions. In a constantly perturbed environment, microbial inhabitants may have been able to adapt to such dynamics and be less responsive to deterministic factors (Santillan et al., 2019). This may explain why stochastic processes dominate the assembly of sediment prokaryotic communities in this study.

Additionally, the specific contributions of subcategories of stochastic and deterministic processes to the prokaryotic community assembly differed across distinct functional areas. Drift, a subcategory of stochastic processes, was 46.4, 31.1, and 40.1% in the multifunctional, commercial, and residential sediments, respectively, (Figure 7B). This could be explained by the alpha diversity variation of sediment communities across the three functional areas. The stochastic process regulates the assembly of a high-diversity prokaryotic community, whereas the deterministic process exhibits a more pronounced prominence when bacterial diversity is lower (Xun et al., 2019). In this study, the highest alpha diversity was found in multifunctional sediments, followed by residential area and commercial area. As a result, the proportion of drift process was the highest in the multifunctional area, while lowest in the commercial area. The neutral model fit well with the prokaryotic communities in all areas, with *R*^2^ values ranging between 0.647 and 0.858 (Figure 7A). These results confirmed that stochastic (neutral) processes played prominent roles in the prokaryotic community assembly of sewer sediment. Comparative analyses among functional areas provide the first informative insights into the patterns in the sewer sediments community assembly. These findings contribute to a better comprehension of the formation and evolution mechanisms of sewer sediment prokaryotic communities, providing significant reference value for future ecological restoration and control strategies.

## 5 Conclusion

In the study, we analyzed the dynamics of prokaryotic communities in the urban sewer in different functional areas using 16S rRNA gene amplicon sequencing. Significant prokaryotic community diversity and composition differences were observed among sediment from typical urban functional areas (multifunctional, commercial, and residential areas), which were mainly correlated with changes in sediment nutrient level and pH. Prokaryotic microorganisms with different functions were gathered in different functional areas. The residential area network has the highest complexity and stability. Potential keystone taxa identified by co-occurrence network analysis also differed among functional areas. The stochasticity process dominated the prokaryotic community assembly. The results of this study will help improve the understanding of the prokaryotic ecology in sewer sediment, providing a foundation for screening of functional microorganisms and supporting a genomic basis for the precise administration of sewer systems in different functional areas. For urban sewer system ecosystems, functional areas may be a new perspective for in-depth analysis of the characteristics of communities. In future work, time-series sampling should be conducted to confirm the observed pattern. In addition, in-depth investigations on sediment prokaryotic communities on a larger scale with greater depth are hoped to further improve our insight into prokaryotic community assembly in sewer sediment.

## Data availability statement

The datasets presented in this study can be found in online repositories. The names of the repository/repositories and accession number(s) can be found below: NCBI - PRJNA1015661.

## Author contributions

JX: Data curation, Formal analysis, Investigation, Methodology, Writing – original draft. KY: Data curation, Formal analysis, Investigation, Writing – review & editing. ZY: Funding acquisition, Investigation, Resources, Supervision, Writing – review & editing. HS: Data curation, Investigation, Software, Supervision, Writing – review & editing. LM: Investigation, Resources, Supervision, Writing – review & editing. DL: Investigation, Project administration, Supervision, Writing – review & editing. HG: Investigation, Supervision, Writing – review & editing. SZ: Investigation, Supervision, Writing – review & editing. DZ: Formal analysis, Investigation, Resources, Supervision, Writing – review & editing.
