# Supplementary material for: Toward an intensive understanding of sewer sediment prokaryotic community assembly and function
Source: Front Microbiol. 2023 Dec 20;14:1327523. doi: 10.3389/fmicb.2023.1327523 (PMC10761402; doi:10.3389/fmicb.2023.1327523)
Supplement: Supplementary file 1 [file Data_Sheet_1.pdf]

Supplementary Material

1 Supplementary Figures and Tables

1.1 Supplementary Figures

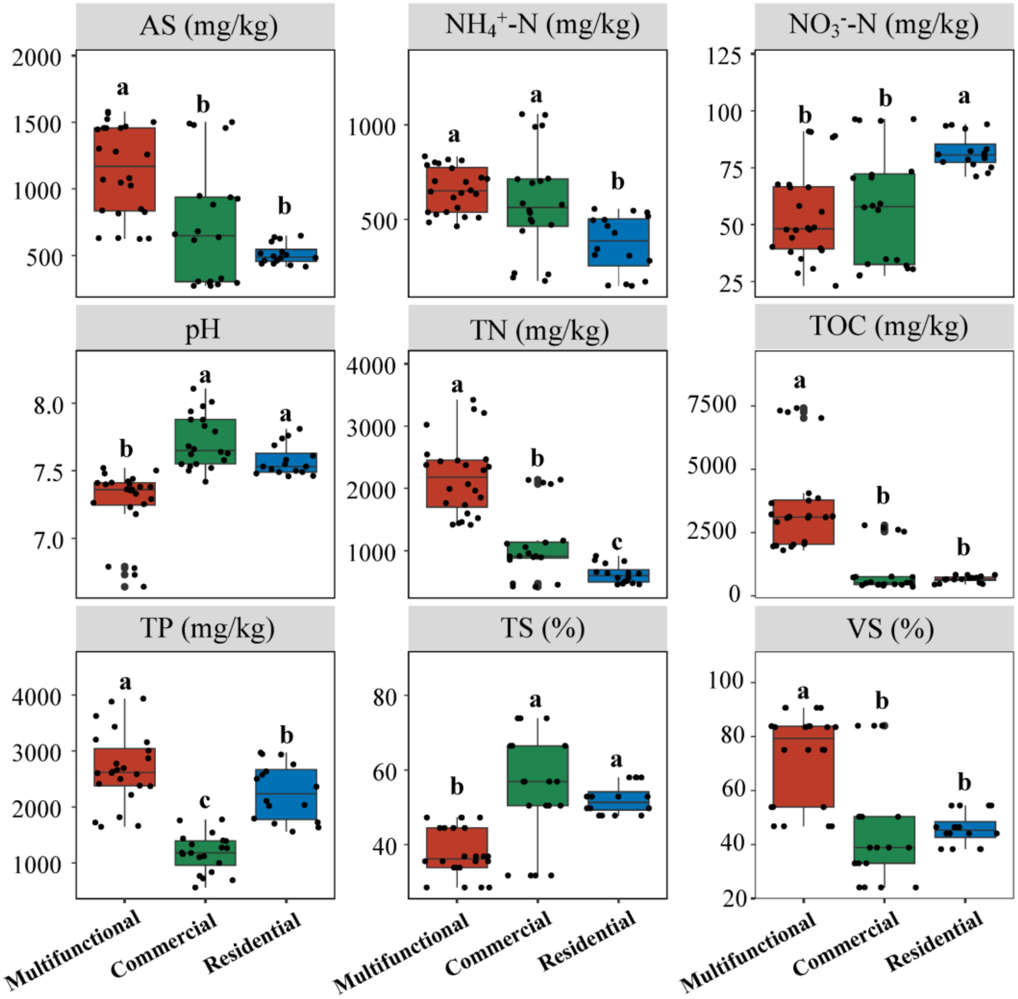

Supplementary Figure 1. Physicochemical properties of sewer sediment from different functional areas. The significance of differences was analyzed using the criteria "a, b, c", with no common superscript indicating significance ($p < 0.05$; multiple comparison with ANOVA tests). TP: total phosphorus; pH: sediment pH; TN: total nitrogen AS: available sulfur; TS: total solid; TOC: total organic carbon; VS: volatile solid; NO_3^--N: nitrate-nitrogen; NH_4^+-N: ammonium-nitrogen.

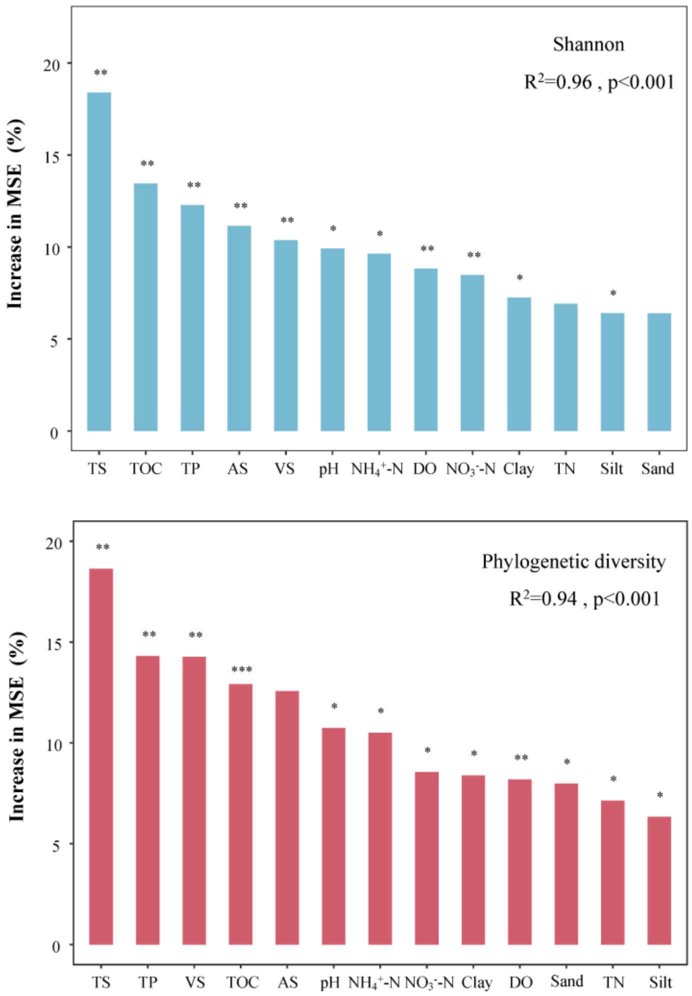

Supplementary Figure 2. Random forest (RF) mean predictor importance (percentage of increase of mean square error) of physicochemical properties as drivers for Shannon indices, and phylogenetic diversity.

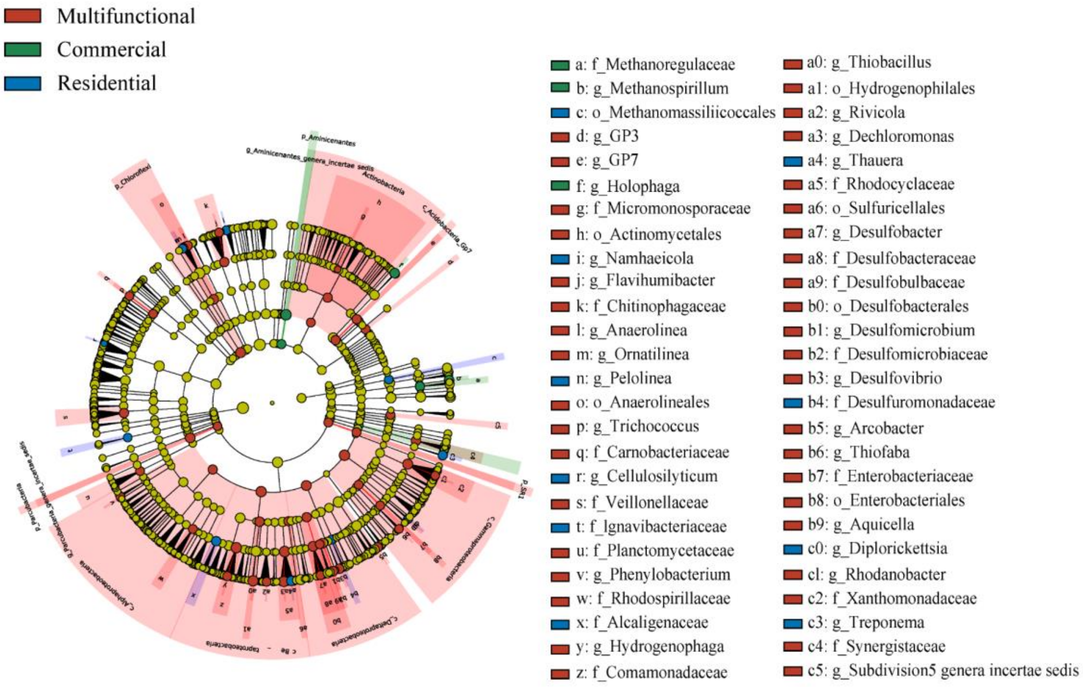

Supplementary Figure 3. Linear discriminant analysis (LDA, threshold 3.2) effect size pipeline (LEfSe) analysis of the significantly discriminant taxa in sewer sediments from different functional area (from phylum to genus, factorial Kruskal-Wallis rank-sum test α=0.05).

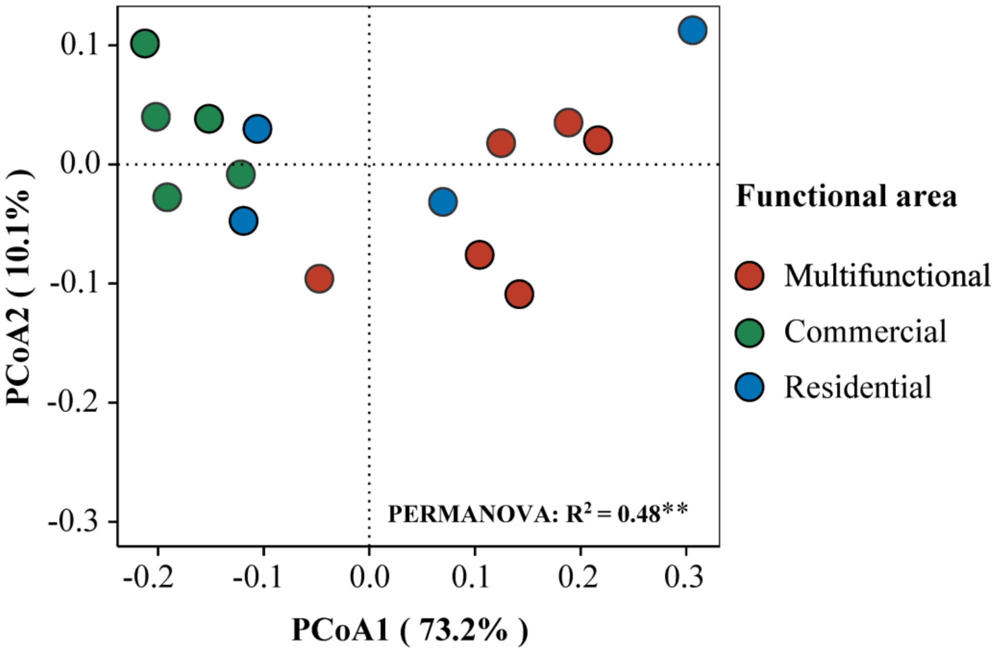

Supplementary Figure 4. Principal coordinate analysis (PCoA) based on Bray-Curtis dissimilarity and PERMANOVA tests shows the variation of functional groups among different functional areas. ***, $p < 0.001$; **$p < 0.01$; *, $p < 0.05$.

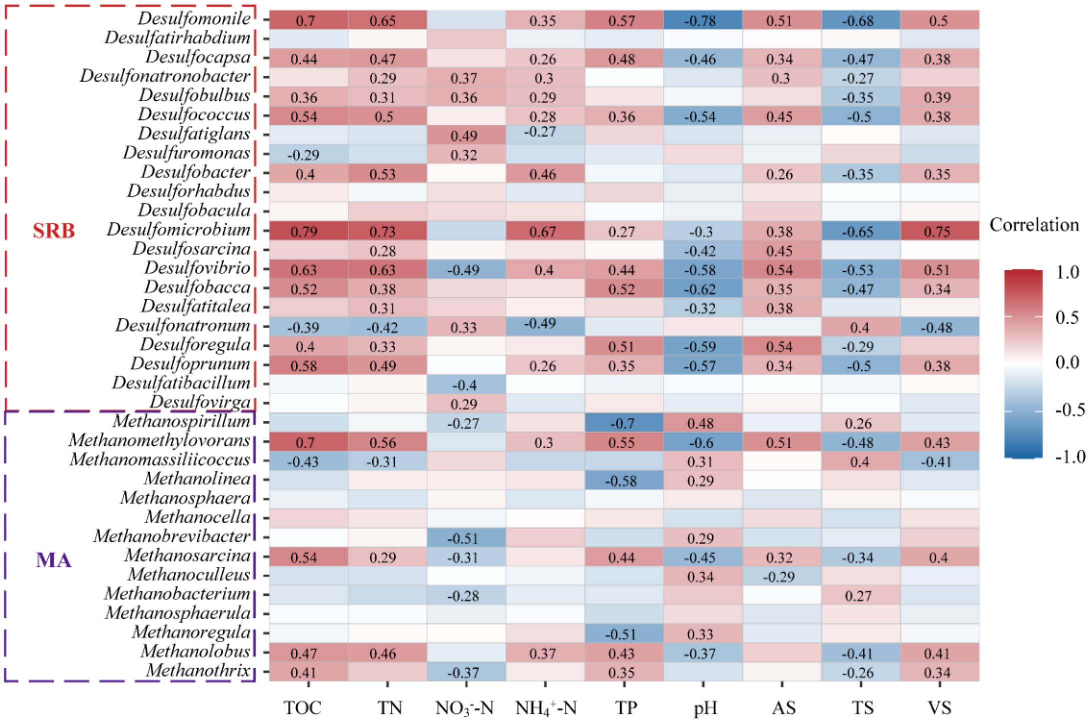

Supplementary Figure 5. Association between the sulfate-reducing bacteria (SRB) and methanogenic archaea (MA) and sewer sediment physicochemical properties. Colors represent Spearman correlations.

1.2 Supplementary tables

Table S1 The specific information of sewer.

	Multifunctional	Commercial	Residential
Collection site type	Comprehensive land-use	Central urban area	Residential land-use
Diameter	300~400 mm	300~400 mm	300~400 mm
Age	~25~35 year	~25~35 year	~25~35 year
Trunk/Branch		Branch sewer	
Transport force		Gravity	
Material		Concrete	

Table S2 The temperature, pH, and dissolved oxygen of sewage.

Functional areas	temperature	pH	dissolved oxygen
Multifunctional	23.65	8.28	0.42
Commercial	25.29	7.81	0.37
Residential	24.43	8.44	0.75

Table S3 Interpretation rate of the conditional effects of each physicochemical property.

Physicochemical property	R^2	p
TP	0.51	0.001
pH	0.42	0.001
TN	0.36	0.001
AS	0.31	0.001
TS	0.31	0.001
TOC	0.25	0.001
VS	0.20	0.002
Silt	0.13	0.019
Sand	0.11	0.049
NO_3^--N	0.10	0.053
Clay	0.06	0.172
NH_4^+-N	0.05	0.21

a: TP: total phosphorus; pH: sediment pH; TN: total nitrogen AS: available sulfur; TS: total solid; TOC: total organic carbon; VS: volatile solid; physical texture (sand, silt, and Clay contents); NO_3^--N: nitrate-nitrogen; NH_4^+-N: ammonium-nitrogen.
